# Some Practical Remarks

**Published:** 1889-03

**Authors:** C. Hering


### SOME PRACTICAL REMARKS.

Cases spoiled by the use of Aconite may often be got right again by giving Sulphur. Arnica is more apt than Aconite to spoil a case. Arnica makes a much more profound impression upon the system than Aconite. Its real culminating action is similar to typhus fever. Brilliant results have frequently been obtained with it in the worst forms of typhus.

Physicians who wear spectacles, and ride long distances in very cold weather, will find protection from freezing of the parts coming in contact with the metal, by bathing the skin with camphor.

*Ranunculus bulb.* is one of our most effective agents for the removal of bad effects from the abuse of intoxicating drinks.

At least one-half of the chronic diseases of women and children are developed by using too much sugar.

*Aconite* is rarely, if ever, of use in *scarlatina*, notwithstanding the "high fever" and the "dry skin," because, instead of the agonizing tossing about of Aconite, the patients are dull and drowsy, the pulse is not hard, etc.

The water treatment: Wet bandages are often of great use in *scarlatina*, but never together with Belladonna. Either the one or the other ought to be omitted. C. HERING.

